# Retraction: Mechanistic modeling and numerical simulation of axial flow catalytic reactor for naphtha reforming unit

**DOI:** 10.1371/journal.pone.0350709

**Published:** 2026-06-03

**Authors:** The PLOS One Editors

The *PLOS One* Editors retract this article [1] due to concerns about compromised peer review and compliance with PLOS policy on Manipulation of the Publication Process.

MR and SS did not agree with the retraction. MP, ATN, and AM either did not respond directly or could not be reached.
